# Carbon-based ruthenium nanomaterial-based electroanalytical sensors for the detection of anticancer drug Idarubicin

**DOI:** 10.1038/s41598-020-68055-6

**Published:** 2020-07-06

**Authors:** S. Irem Kaya, Sevinc Kurbanoglu, Ejmer Yavuz, Sibel Demiroglu Mustafov, Fatih Sen, Sibel A. Ozkan

**Affiliations:** 1 Department of Analytical Chemistry, Faculty of Pharmacy, Ankara University, Ankara, Turkey; 2 Sen Research Group, Biochemistry Department, Faculty of Arts and Science, Dumlupınar University, Evliya Celebi Campus, 43100 Kutahya, Turkey

**Keywords:** Electrochemistry, Bioanalytical chemistry

## Abstract

In this work, a novel nanosensing platform was suggested based on ruthenium for the sensitive determination of Idarubicin anticancer drugs. Ruthenium/Vulcan carbon-based nanoparticles were synthesized ultrasonication method and then characterized by transmission electron microscopy (TEM), X-ray photoelectron spectroscopy (XPS), and X-ray diffraction (XRD). The mean particle size of the nanoparticles calculated by the TEM analysis was found to be 1.98 nm ± 0.29 nm, and the Ru nanoparticles were mostly dispersed on the support material. Glassy carbon electrode (GCE) surface was modified with Ruthenium/Vulcan carbon-based nanomaterials (Ru@VC), and characterization of the nanosensor was performed using electrochemical impedance spectroscopy and cyclic voltammetry. The limit of detection (LOD) and limit of quantification (LOQ) values were found as 9.25 × 10^–9^ M and 2.8 × 10^–8^ M in buffer samples. To demonstrate the applicability and validity of developed nanosensor, it was used for the determination of Idarubicin in Idamen^®^ IV (10 mg/10 mL vial) and human serum sample. The results of recovery studies showed that the Ru@VC/GCE nanosensor was free from excipient interferences in the dosage forms of injection, and it can be successfully applied to biological samples.

## Introduction

Cancer chemotherapy with antineoplastic drugs is based on eliminating cancer cells by killing them or inhibiting cell growth and minimizing the effect on healthy cells^1,2^. Cancerous cells have one important difference when compared to healthy cells: While normal cells experience a programmed cell death process (apoptosis) cancerous cells do not^1,2^. At this point, the main aim of antineoplastic drugs is to focus this difference and specifically affect cancer cells, but unfortunately, most drugs target not only malignant cells but also healthy cells with proliferative properties such as bone marrow and hair follicle cells resulting with adverse effects such as alopecia, anemia, and thrombocytopenia etc.^1, 2^. All cytotoxic drugs which are used for cancer treatment affects deoxyribonucleic acid (DNA) synthesis, and they can be divided into various classes based on their action site of the cell cycle: Alkylating agents (e.g., Bendamustine, carboplatin, ifosfamide, melphalan), Antimetabolites (e.g., Fludarabine, fluorouracil, methotrexate, pemetrexed), Antibiotics (e.g., Bleomycin, doxorubicin, epirubicin, idarubicin), Mitotic inhibitors (e.g., Docetaxel, paclitaxel, vincristine), Monoclonal antibodies (e.g., Cetuximab, bevacizumab, rituximab), Signal transduction inhibitors (e.g., Erlotinib, imatinib, sorafenib), Steroid hormones and their antagonists (e.g., Lepurolide, raloxifene, tamoxifen), Immunosuppressive agents (e.g., Cyclosporine, tacrolimus), Others (e.g., Asparaginase, etoposide)^1,2^.

Idarubicin (IDA), which is a cytotoxic antibiotic, is chemically designated as (1S,3S)-3-acetyl-3,5,12-trihydroxy-6,11-dioxo-1,2,3,4,6,11-hexahydronaphthacen-1-yl 3-amino-2,3,6-trideoxy-α-l-lyxo-hexopyranoside and it is used for the treatment of different types of cancers like leukemia, myeloma and hematological diseases^3,4^. Various chemotherapy protocols involve IDA and are still in use, such as cytarabine + fludarabine + IDA, dexamethasone + IDA^5^. IDA is simply described as a more lipophilic, synthetic analog of doxorubicin, which has an anthracycline structure, and its mechanism of action is intercalating among DNA base pairs and inhibiting topoisomerase II^2,4^.

Considering all of these, developing a new, fast, low cost and a practical sensor to the determination of IDA in pharmaceutical dosage forms and biological fluids is important. Amongst a wide range of different techniques for the determination of compounds, electroanalytical methods are one of the most advantageous and widely used techniques due to their sensitivity, accuracy, reliability, and low cost^6–13^.

The GCE is the most widely employed electrode in electrochemical drug analysis due to its advantageous electrical, mechanical, and physical properties such as chemical inertness, wide potential range, and compatibility^4,6^.

In electrochemical events, the electrode acts as a catalyst in charge transfer reactions. Reactions occur at the constant voltage on the surface of the active electrode^14–16^. The most important parameter in electrocatalytic processes is to increase the active surface area of the electrode^17–20^. The ultimate goal is to enhance this surface area and to increase the reaction rate at a lower voltage. Therefore, elements that increase the charge transfer in the active surface area of the electrode and conductive materials are used to support these elements^21–26^. Catalyst supports have a significant impact on catalyst performance in order to provide usable surface shape, particle size, electroactive zone, and stability. Vulcan carbon is one of the most widely used carbon support materials with excellent electron transfer capacity, active surface area, and bonding to the electrode surface. Vulcan carbon increases oxidation through active metal particles adsorbed^27–31^. The ruthenium element has the effect of increasing electronic conductivity. It facilitates electron movements in redox reactions and has a positive effect on the separation of electrons from the charge center. This provides increased selectivity and sensitivity in electrochemical studies^32^. Ruthenium particles impregnated with pores on the Vulcan carbon surface have a catalytic effect. Most of the present studies are based in the literature on chromatographic analysis like high-performance liquid chromatography (HPLC)^33,34^, reverse-phase liquid chromatography^35,36^; phosphorescence^37^; fluorescence spectroscopy^3,38^; capillary electrophoresis^39^, ultraviolet–visible (UV–VIS) spectrophotometry^40^ and combined techniques such as liquid chromatography-ultraviolet spectroscopy, mass spectroscopy (LC-UV, MS) and LC–MS-Time of flight (TOF)^41^. Hence, there are not many studies; only two studies exist for the determination of Idarubicin using electrochemical nanosensors. One of them is again from our group, where we used carbon nanotubes modified glassy carbon and edge plane graphitic electrode. In that study, we achieved the detection of Idarubicin up to 10^–8^ M level^4^. In another study, TiO_2_ nanoparticles and carbon nanofibers were used by Arkan et al.^42^. Therefore, this work aims to monitor the inorganic nanomaterial platform based on ruthenium for the sensitive and rapid determination of Idarubicin. For this aim, a novel nanomaterial (Ru@VC) is synthesized, and a new and more sensitive electrochemical nanosensor using a new nanomaterial for investigation of the electrochemical behavior and determination of IDA in pharmaceutical dosage forms and human serum sample is achieved.

## Experimental

### Apparatus

Electrochemical impedance spectroscopy (EIS) measurements were done using a Metrohm Autolab Potentiostat/Galvanostat device. PalmSens4 Potentiostat/Galvanostat/Impedance Analyzer was used for all the other voltammetric measurements for IDA. The working conditions of cyclic voltammetry (CV) were: initial potential, − 0.2 V; scan rate, 100 mV/s; potential step, equilibration time, 5 s; 2 mV; N scans, four and current range, 1,000 µA. In the measurements of differential pulse voltammetry (DPV), initial potential, − 0.2 V; pulse amplitude, 50 mV; scan rate, 20 mV/s; pulse width, 200 ms; potential step, 20 mV; final potential, 1.2 V; current range, 10 µA, and equilibration time, 5 s were utilized. In the measurements of adsorptive stripping differential pulse voltammetry (AdSDPV), scan rate, 20 mV/s; pulse width, working potential, − 0.2 V–1.2 V; pulse amplitude, 50 mV; 200 ms; equilibration time, 5 s; potential step, 20 mV; current range, 10 µA, deposition time 120 s, and deposition potential 0 V were utilized. In addition, the three-electrode system consists of a GCE (BAS, 3 mm, diameter) as the working electrode, a Pt wire as the auxiliary electrode, and an Ag/AgCl electrode as the reference electrode. In the course of the experiment, prior to each measurement, the GCE was polished on a damp polishing cloth (BAS velvet polishing pad) by an aqueous slurry of alumina (0.01 μm, Metkon ALU-MIK) until a mirror-like finish was obtained. Before each AdSDPV measurement for the electrochemical cleaning of the modified glassy carbon electrode, 20 CV cycles were applied. A pH meter Model 538 (WTW, Germany) was utilized for the pH measurements with a combined electrode with an accuracy of ± pH. To the drying process, the Nuve EV 018 vacuum oven was employed.

### Reagents and chemicals

Idamen^®^ IV 10 mg/10 mL vial and IDA were provided by pharmaceutical companies in Turkey. Sodium phosphate monobasic dihydrate, sulphuric acid, acetic acid, phosphoric acid, disodium phosphate, sodium acetate trihydrate, ruthenium(III) chloride (RuCl_3_), DMAB [4-(dimethylamino) benzaldehyde], Vulcan^®^ XC72R, human serum samples, methanol, and ethanol were obtained from Sigma-Aldrich. Also, the water was processed to be analytical grade using the Millipore water treatment system in all experiments. Under the argon atmosphere, 99.5% tetrahydrofuran (THF) from Merck was made ready.

### Preparation of solutions

The stock solution of 1 × 10^−3^ M IDA was prepared in methanol. In order to prepare working solutions of IDA with different concentrations, the stock solution diluted with desired pH buffer solutions containing 20% methanol. For investigating the pH effect on the electrochemical studies; 0.5 M H_2_SO_4_ (pH 0.3) and 0.1 M H_2_SO_4_ (pH 0.5) solutions, phosphate buffer solutions (pH 1.5, 2.5, 3, 6.02, 6.5, 7.0, and 8.0), acetate buffer solutions (pH 3.7, 4.7, and 5.7) were utilized as supporting electrolyte. All supportive electrolyte solutions were prepared from a Millipore Milli-Q device utilizing double-distilled water and kept in a refrigerator at 4 °C.

### Synthesis and characterization of Ru@Vc nanomaterial

Ruthenium and Vulcan carbon were taken in a 1: 1 ratio (w/w) of 30 mg, ethanol was used as the solvent. The mixture was ultrasonicated for 30 min in an ultrasonic bath. Stirred under nitrogen for half an hour, 0.184 mg DMAB was added. Stirred for 12 h, then washed by water and ethanol and dried. TEM, XPS, and XRD analyses were employed to determine the morphological, structure, and chemical composition of Ru@VC nanomaterials. TEM analysis of Ru@VC nanomaterials was performed utilizing a JEOL 200 kV TEM microscope. A Specs spectrometer was employed for XPS analysis employing Kα lines of Mg (1,253.6 eV, 10 mA) as an X-ray source.

XRD was done utilizing a Panalytical Empyrean diffractometer with Ultima + theta-theta high-resolution goniometer, an X-ray generator (Cu K radiation, λ = 1.54056 Å) with 45 kV and 40 mA operating conditions.

### Ru@VC based nanosensor preparation

For the preparation of Ru@VC suspension, firstly, 1 mg of Ru@VC was distributed in 1 mL distilled water. Later, utilization of the ultrasonic bath, the suspension was ultrasonicated for 2 h. Before GCE was modified, the electrode surface was polished on a polishing cloth with alumina slurry, then washed by distilled water and dried with air. Lastly; the nanomaterial amount optimization study was performed by dropping various volumes of Ru@VC suspension (1 µL, 3 µL, 5 µL, 7 µL, and 10 µL) onto the surface of the electrode and drying in a vacuum oven.

### IDA analysis from dosage forms and human serum samples

Idamen^®^ IV 10 mg/10 mL vial contains 10 mg Idarubicin Hydrochloride for injection. The working solution of IDA with 20% methanol was prepared by dilution of the stock solution to 2.5 × 10^–7^ M with pH 1.5 phosphate buffer solution. In order to prepare standard serum solution, 1 mL of IDA from 1 × 10^−3^ M stock solution of IDA, 3.6 mL serum, and 5.4 mL acetonitrile were put together in a 10 mL centrifuge tube, and this mixture was centrifuged for 30 min at 3,500 rpm. After the centrifuge process and separation of protein residues and supernatant, the supernatant was taken and used for serum sample analysis. For further electrochemical analysis with serum samples, working solutions were prepared using supernatant as serum stock solution with methanol and phosphate buffer solution at pH 1.5 and were analyzed under optimum conditions. The recovery studies were performed using a standard addition method to prove the reliability, applicability, and accuracy of the proposed nanosensor from real samples.

## Results and discussion

### Synthesis of Ru@VC nanomaterial

**Figure 1:**
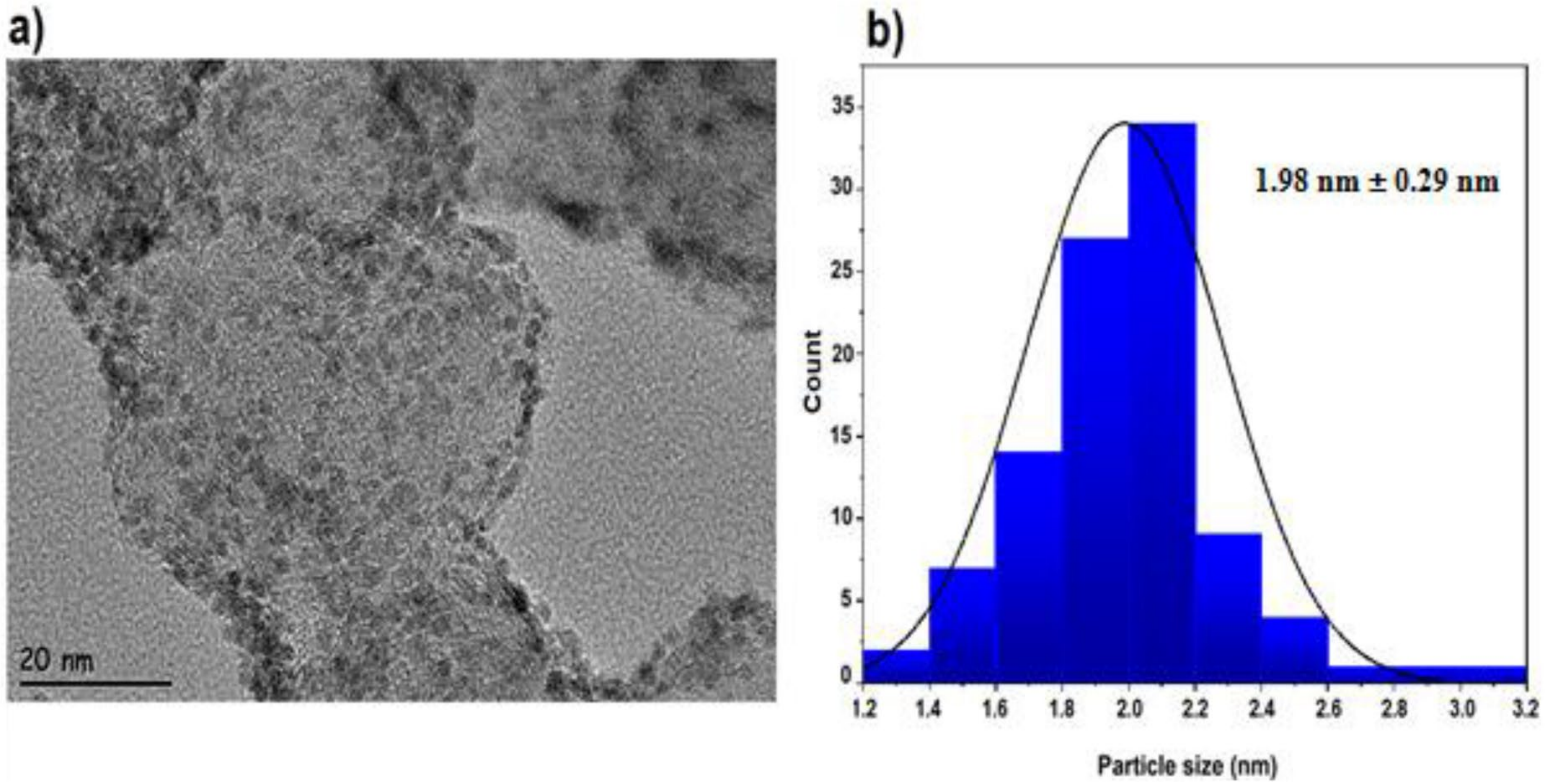
(**a**) TEM image and (**b**) particle size histogram of Ru@VC nanomaterials.

The distribution of Ru metals on VC support materials and the particle size of the formed Ru@VC nanomaterials were investigated by TEM analysis. Figure 1a indicates well and almost homogenous distributions of the metals on the support materials. Figure 1a also shows the negligible level of particle agglomeration has been obtained on the surface of Vulcan carbon supports. The average particle size was calculated utilizing an image analyzer (ImageJ software). About 100 particles were employed for the calculation of the size distribution. As shown in Fig. 1b, the average particle size of Ru@VC nanomaterials was found as 1.98 nm ± 0.29 nm that result has a good agreement with the previous studies^43–45^.

**Figure 2:**
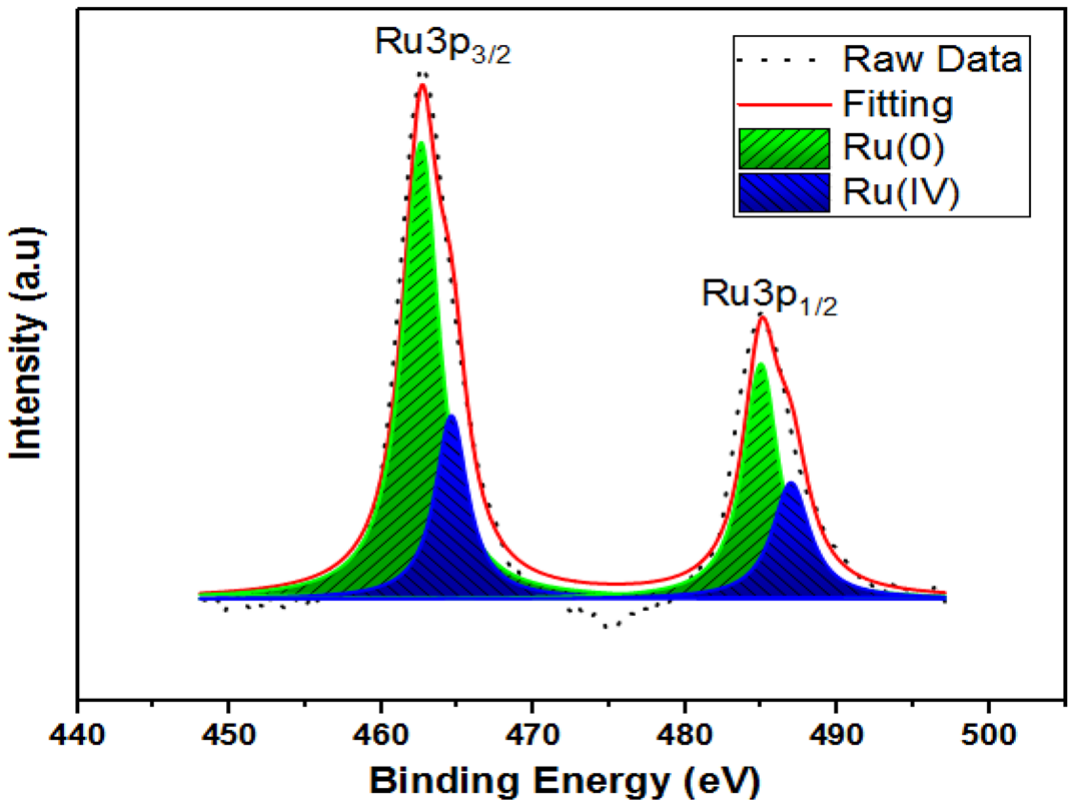
XPS pattern of ruthenium in Ru@VC nanomaterials.

XPS analysis also studied the oxidation state of Ru@VC nanomaterials according to the Gaussian–Lorentzian process. Figure 2 indicates the XPS pattern of Ru and shows the main peaks at 462.60, 484.97 eV assigned to Ru^0^ and 464.60, 486.96 eV assigned to Ru^4+^, respectively. Additionally, the binding energies of 462.60 eV and 486.96 eV were observed in the Ru 3p_3/2_ and 3p_1/2_ orbital XPS spectra of Ru@VC nanomaterials^46–48^.

**Figure 3:**
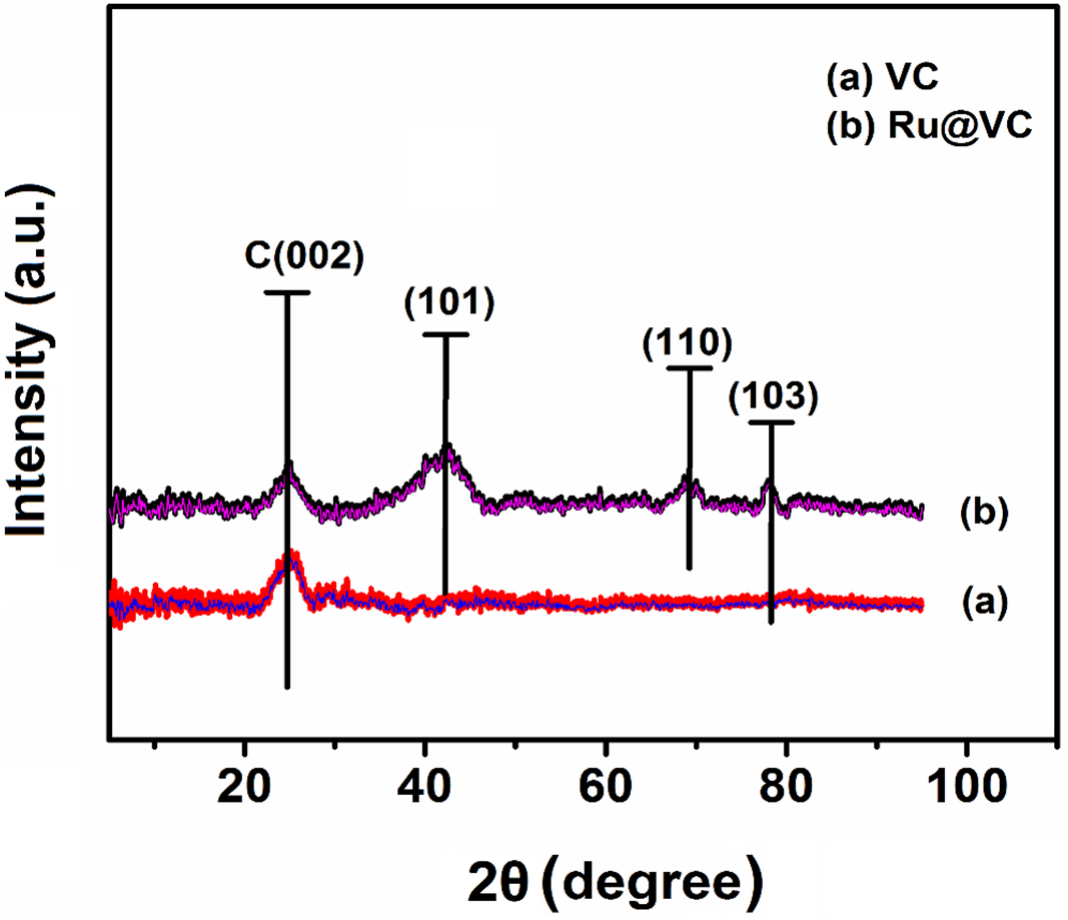
XRD patterns of Ru@VC nanomaterials.

The crystal structures of the Vulcan carbon and synthesized Ru@VC nanomaterials were characterized by utilizing XRD, the results of which are displayed in Fig. 3. The characteristic peaks appearing at 2θ values of 26° and 42.3° may be ascribed to Vulcan carbon corresponding to (002) and (101) crystal plane, respectively^49^. From Fig. 3, it detected that the XRD pattern of Ru@VC nanomaterials displays the diffraction peaks at 68.32°, and 77.2° represented by (110) and (103), the crystal planes of ruthenium, respectively. Furthermore, the 101 crystal planes of face-centered cubic (fcc) structures of ruthenium were detected at around 2θ = 42.3°^50,51^.

### Electrochemical characterization of Ru@VC/GCE

**Figure 4:**
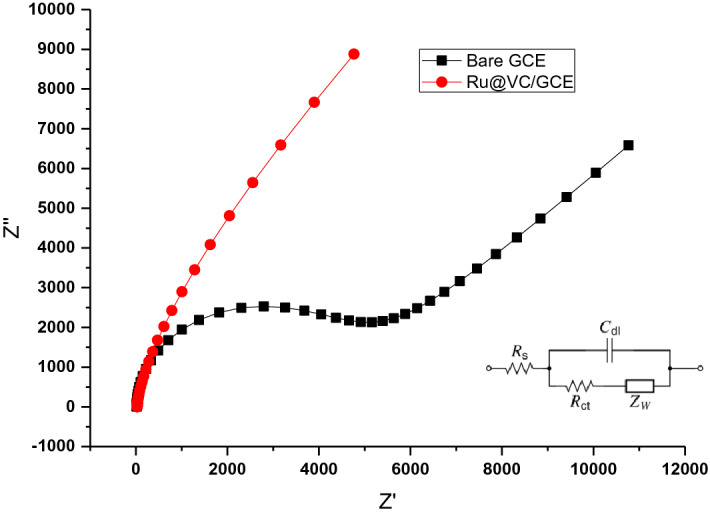
Nyquist plot of bare GCE and Ru@VC/GCE. Inset: The Randles equivalent circuit model (R_s_: solution resistance, C_dl_: constant phase element, R_ct_: the charge transfer resistance, Z_w_: Warburg impedance), which was used to fit the data.

EIS is a significant electrochemical method for explaining differences between unmodified and modified electrodes in terms of conductivity or impedance for oxidation or reduction processes^52^. In this work, EIS measurements were performed using 5 mM K_3_[Fe(CN)_6_] solution as the redox probe in order to describe and compare electrochemical characteristics of bare GCE and Ru@VC/GCE. The results are given in Fig. 4 in the form of a Nyquist plot. The parameters determined after fitting the results to the Randles equivalent circuit model are listed in Table 1. As it is displayed in Fig. 4, the EIS Nyquist plot of Ru@VC modified GCE has a smaller semicircle compared to bare GCE. These results indicate that the modified GCE has better electronic conductivity and enhances electron transfer kinetics compared to bare GCE. Besides, the surface of Ru@VC/GCE has faster electron transfer and decreased charge transfer resistance.

**Table 1:** Parameters calculated from EIS.

	R_e_ (Ω)	R_ct_ (Ω)	CPT (µF)
GCE	17.0	4,430	1.98
Ru@VC/GCE	29.2	0.114	40.4

### pH effect on electrochemical studies

**Figure 5:**
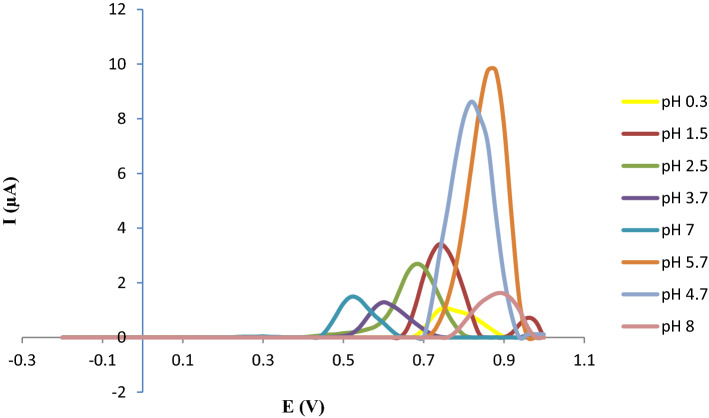
Differential pulse voltammetric responses of Ru@VC/GCE at different pH with scan rate 50 mV s^−1^.

In order to investigate the influence of the pH on the oxidation peak potentials and peak currents of IDA, H_2_SO_4_ solutions, acetate, and phosphate buffer solutions pH values ranging from 0.3 to 8 were used by DPV using Ru@VC/GCE (Fig. 5). The relationship among peak potential (*Ep*) and pH can be described by the Eq. (1) as follows:

1 $$Ep \left(\text{mV}\right)= - 55.134 pH + 802.27 \;using \;Ru@VC/GCE\; (r = 0.979; DPV)$$

The pH increase resulted in a shift of E*p* to less positive values. While E*p* demonstrated a linear response against pH, it also showed that E*p* was pH-dependent. Furthermore, the slope value of the equation above is close to the theoretical value of − 59 mV, and it suggests that equal numbers of protons and electrons are involved in the rate-determining steps^53^.

IDA's peak current (I_p_) reached the max peak when the pH was increased from 0.3 to pH 1.5. Starting with pH 4.7, peaks of modification material interfered with IDA peaks and caused inconsistent results. Therefore, pH 1.5 phosphate buffer was chosen as the optimal pH value and was utilized in additional measurements.

### Scan rate effect on electrochemical studies

**Figure 6:**
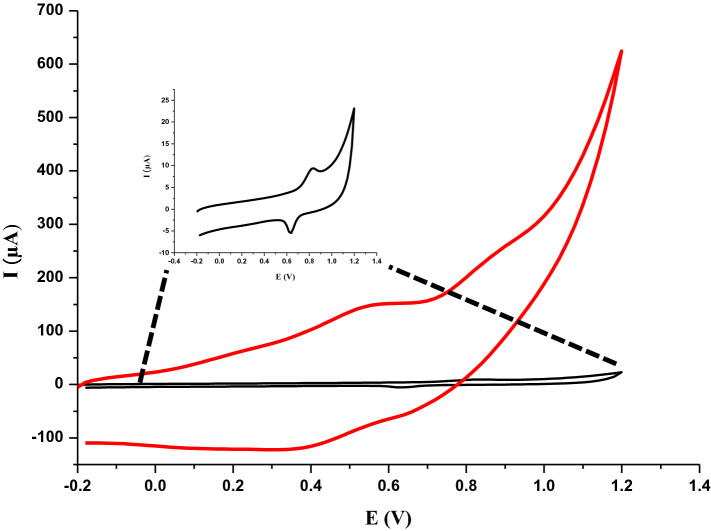
Cyclic voltammograms of 5 × 10^–6^ M IDA on bare GCE and Ru@VC/GCE.

The electrochemical behavior of 5 × 10^–6^ M IDA was studied at optimum pH, which is in phosphate buffer (pH 1.5), on bare GCE and Ru@VC/GCE by CV (Fig. 6). Same as in our previous study^4^, a well-defined, oxidation peak was obtained around 700 mV with bare GCE, whereas Ru@VC/GCE showed a more broad peak for the same concentration of IDA with very high current values.

The scan rate studies were performed to understand the oxidation behavior of IDA on Ru@VC/GCE, whether if it is diffusion or adsorption controlled. 5 × 10^–6^ M IDA was investigated at Ru@VC/GCE using CV in phosphate buffer pH 1.5 in the range of 5 to 1,000 mV/s. The decrease of scan rate resulted in the shift of E*p* to lower potential values and the decrease of I_*p*_:

2 $$Ep (V) = 0.0771\log\upsilon + 0.8815 \quad r= 0.992$$

Equation (3) can represent the relationship between I_p_ and *v*. The linearity of I_p_ vs. *v*indicates that the oxidation mechanism is adsorption controlled.

3 $$Ip(\upmu A) = 0.0072\upsilon-0.2785 \quad r= 0.969$$

Moreover, a log I_*p*_ vs. log *v* graph was also obtained to understand the process deeply. If the correlation coefficient of the log I_*p*_ vs. log *v* is close to 0.5, that indicates the diffusion-controlled electrode process, and if it is close to 1, that indicates the adsorption controlled electrode process^54–56^.

4 $$\log Ip =0.6504 \log v-1.1519 \quad r= 0.992$$

**Figure 7:**
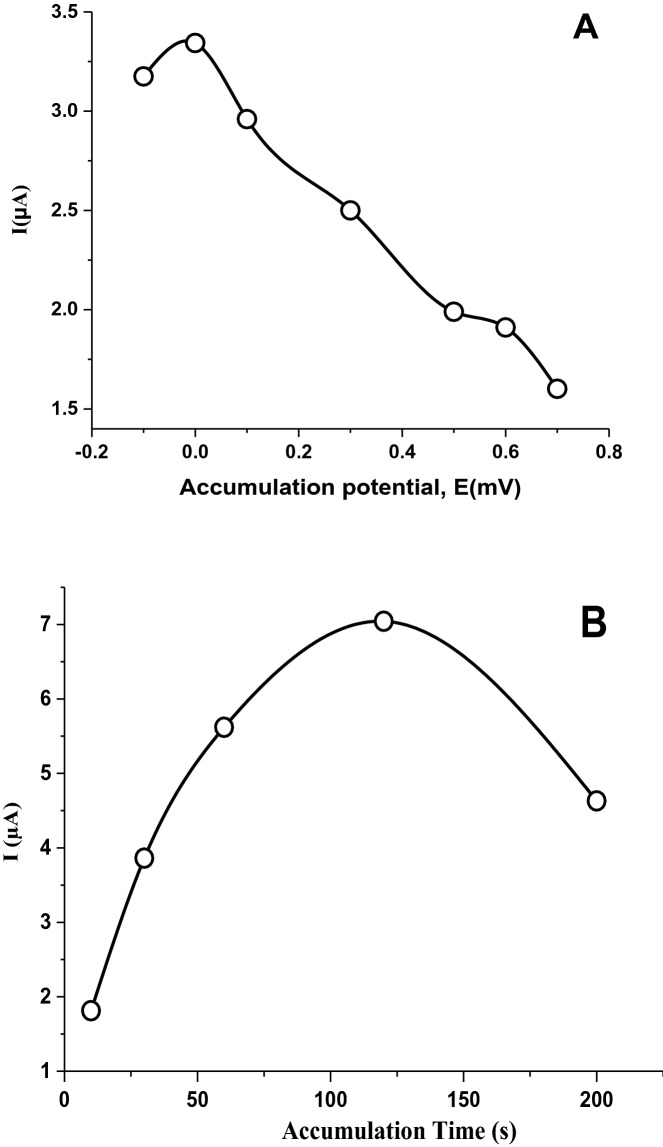
(**A**) Accumulation potential effect on the peak currents with 30 s of accumulation time. (**B**) Accumulation time effect on the peak currents, with an accumulation potential at 0.0 mV for 5 × 10^–6^ M IDA in pH 1.5 phosphate buffer utilizing the AdSDPV method.

It can be understood from Eq. (3) that the nature of the electrode process is adsorption controlled. Therefore, deposition potential and deposition time were further optimized. In the adsorption process, the optimal deposition time and potential were studied using 5 × 10^–6^ M IDA in pH 1.5 phosphate buffer using the AdSDPV methods with Ru@VC/GCE nanosensor. Amongst different potential values between − 0.1 and 0.7 V, and different time values between 10 and 300 s; using 0 V accumulation potential and 30 s accumulation time, the highest oxidation peak of IDA was acquired, and these values were chosen as optimum conditions for further experiments (Fig. 7).

### Modification effect of nanomaterial on the electrochemical response

**Figure 8:**
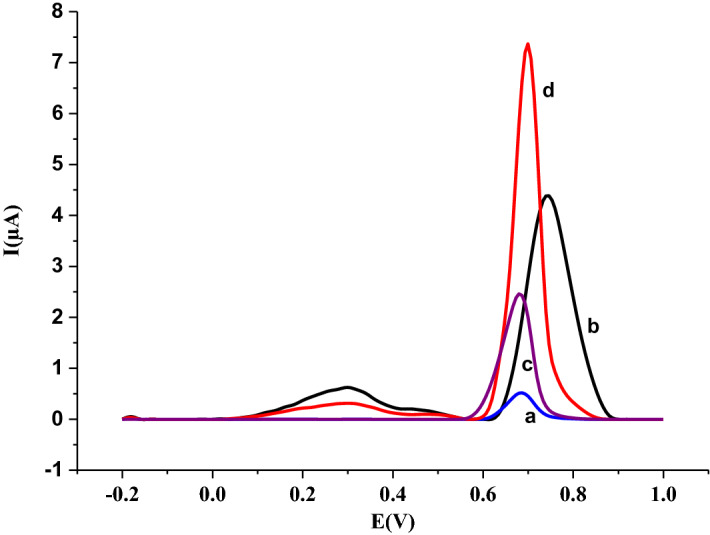
(**a**) Voltammogram of 5 × 10^–6^ M IDA in pH 1.5 phosphate buffer using a) bare GCE by DPV method (**b**) Ru@VC/GCE by DPV method (**c**) bare GCE by AdSDPV method with 0.0 mV accumulation potential, 30 s accumulation time (**d**) Ru@VC/GCE by AdSDPV method with 0.0 mV accumulation potential, 30 s accumulation time.

For the modification effect studies, firstly, Ru@VC suspension was prepared by dispersing nanomaterials in distilled water (1 mg/mL, ultrasonication for 2 h) utilizing an ultrasonic bath. Prior to the modification, the bare GCE’s surface was polished with alumina slurry on a polishing cloth, cleaned by distilled water and dried. After that, 5 µL of Ru@VC suspension was dropped onto the surface of bare GCE and dried in a vacuum oven. For the comparison of bare and modified GCEs, the voltammetric behavior of 5 × 10^–6^ M IDA was studied in phosphate buffer at pH 1.5 by DPV using first bare GCE and then Ru@VC/GCE. When the acquired voltammograms examined, it was understood that with Ru@VC modification, the peak current of IDA (Fig. 8b) increased 9 times compared to the bare GCE (Fig. 8a). It indicates that the oxidation of IDA is easier on Ru@VC/GCE than bare GCE. After performing scan rate studies and determining adsorption-controlled processes for the oxidation of IDA, 5 × 10^–6^ M IDA was studied in pH 1.5 phosphate buffer by AdSDPV using bare GCE (Fig. 8c) and Ru@VC/GCE (Fig. 8d). There exist nearly 16 times an increase in the response of IDA. Obtained voltammograms which show the modification effect and deposition effect were compared in Fig. 8.

### Optimization of nanomaterial amount

**Figure 9:**
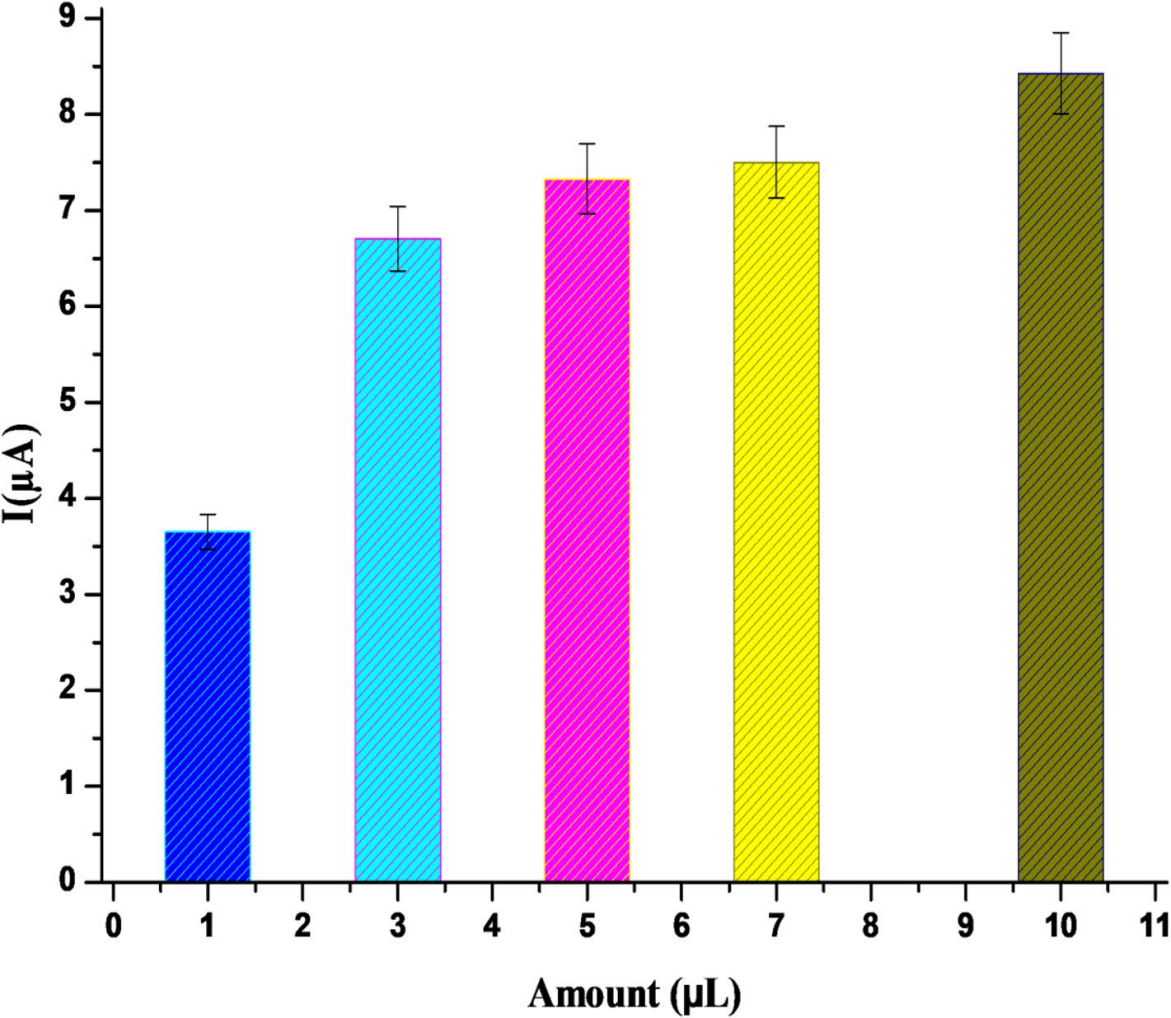
Effect of Ru@VC amount on IDA response using Ru@VC/GCE by AdSDPV method with 0.0 mV accumulation potential, 30 s accumulation time.

For investigating the effect of nanomaterial amount¸ 1 µL, 3 µL, 5 µL, 7 µL, and 10 µL of Ru@VC suspension was dropped to the surface of the electrode, drying in a vacuum oven. The voltammetric behavior of different Ru@VC suspension amount was studied using 5 × 10^–6^ M IDA by AdSDPV (Fig. 9). The results showed that the highest I_*p*_ was obtained with 10 µL of Ru@VC. On the other hand, it was hard to maintain a steady drop with 10 µL of nanomaterial on the electrode surface, and it caused a longer drying time, which makes this higher amount is a non-optimal condition. Thus, the second-best option, 7 µL, was preferred as the optimal nanomaterial amount and used in the subsequent studies.

### Analytical characterization and validation of the nanosensor

**Figure 10:**
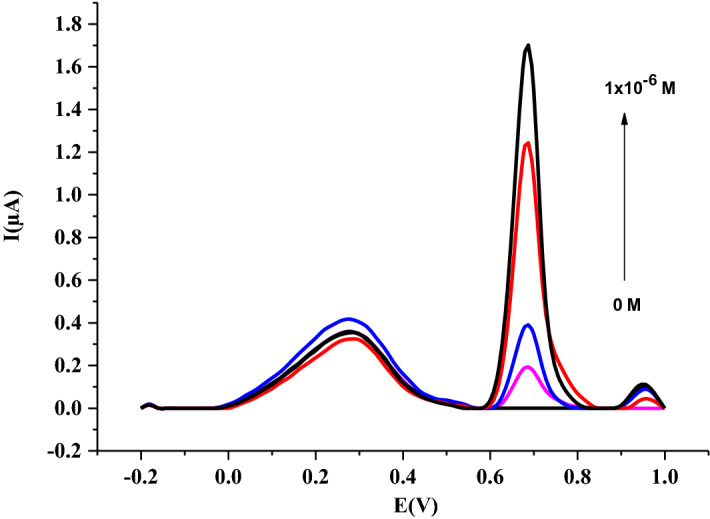
AdSDPV voltammograms at Ru@VC/GCE in phosphate buffer at pH 1.5 with 0.0 mV accumulation potential, 30 s accumulation time.

Quantitative analysis of IDA was performed using Ru@VC/GCE sensor by the AdSDPV method under the selected optimum conditions at 0.0 mV accumulation potential, 30 s accumulation time. The calibration graph of I_*p*_ vs. concentration of IDA gave a linear response among 5 × 10^–8^ M and 1 × 10^–6^ M (Fig. 10). The data obtained from this graph was listed in Table 2. The AdSDPV method calibration equation was given below:

**Table 2:** Regression data of the calibration graphs for IDA on Ru@VC/GCE.

	IDA in buffer solution	IDA in serum
Linearity range (M)	5 × 10^–8^ M–1 × 10^–6^ M	5 × 10^–8^ M–2.5 × 10^–7^ M
Slope (µA M^−1^)	1514.3	322.33
SE of slope	60.81	33.44
Intercept (µA)	7.848 × 10^–2^	3.67 × 10^–2^
SE of intercept	2.834 × 10^–2^	5.546 × 10^–3^
Correlation coefficient (*r*)	0.998	0.991
LOD (M)	9.25 × 10^–9^	7.40 × 10^–9^
LOQ (M)	2.8 × 10^–8^	2.24 × 10^–8^
Reproducibility of peak current (RSD %)	0.153	1.432
Reproducibility of peak potential (RSD %)	0.801	0.846

5 $$Ip \left(\upmu A\right)= 1514.3 C\left(M\right)+ 0.0785 \quad r=0.998$$

The values of LOD and LOQ were determined to utilize the following equations;

$$LOD = {33} {\text{ s}}/{\text{m}}$$

$$LOQ = {10}{\text{ s}}/{\text{m}}$$

where *s* is the standard deviation’s response, and *m* is the calibration curve’s slope ^57–59^.

The values of LOD and LOQ were calculated as 9.25 × 10^–9^ M and 2.8 × 10^–8^ M, as summarized in Table 2 with the reproducibility of peak current and potential. IDA was also determined in the human serum sample, and the calculated results were given in Table 2. The linear range was obtained between 5 × 10^–8^ M and 2.5 × 10^–7^ M IDA in human serum samples with the LOD and LOQ values of 7.24 × 10^–9^ M and 2.19 × 10^–8^ M, respectively. When we compare obtained results with the literature, better LOD responses were received from our previous study, where we used multiwalled carbon nanotubes^4^ and from Arkan et. where they used TiO_2_ nanoparticles and carbon nanofibers^42^ that are summarized in Table 3.

**Table 3:** Comparison of studies for IDA detection.

Electrode	Method	Linear range (M)	LOD (M)	LOQ (M)	Ref
MWCNT-GCE	AdSDPV	9.36 × 10^–8^–1.87 × 10^–6^	1.87 × 10^–8^	7.49 × 10^–8^	^4^
MWCNT-EPPGE	AdSDPV	9.36 × 10^–8^–9.36 × 10^–7^	3.75 × 10^–8^	9.36 × 10^–8^	^4^
TiO_2_-CNF/CPE	CV	1.2 × 10^–8^–1.0 × 10^–5^	3.0 × 10^–9^	–	^42^
Ru@VC/GCE	AdSDPV	5 × 10^–8^ M–1 × 10^–6^ M	9.25 × 10^–9^	2.80 × 10^–8^	This work

*CNF* carbon nanofibers, *CPE* carbon paste electrode, *EPPGE* edge plane pyrolytic graphite electrode, *MWCNT* multiwalled carbon nanotubes, TiO_2_ titanium dioxide nanoparticles.

### Application to pharmaceutical dosage forms and human serum

To assess the applicability and validity of developed nanosensor, it was used for the determination of IDA in Idamen^®^ IV (10 mg/10 mL vial) and human serum samples using the standard addition method. Idamen^®^ IV (10 mg/10 mL vial) contains 10 mg of Idarubicin Hydrochloride as an active substance and water for injection as excipients^60^. The recovery results for Idamen^®^ IV and human serum samples were listed in Table 4. The results indicated that the proposed nanosensor could be successfully applied to pharmaceutical dosage forms and real samples with acceptable precision and accuracy results.

**Table 4:** Results of recovery for the pharmaceutical dosage form Idamen^®^ and human serum sample.

Parameters	Idamen^®^	Serum
Labeled claim (mg)	10.00	–
Amount found (mg)	10.04	–
RSD %	0.85	–
Bias %	0.04	–
Added (mg)	5.00	5.00
Found (mg)	5.02	5.11
Average recovered %	100.41	102.25
RSD % of recovery	0.68	0.65
Bias %	− 0.41	− 2.25

## Conclusion

The current study paves ways to investigate voltammetric behavior to determine the anticancer drug IDA in human serum samples and a pharmaceutical dosage form by AdSDPV of a novel Ru@VC/GCE nanosensor that promises practical applications. The synthesized ruthenium/Vulcan carbon-based nanomaterials were characterized by XPS, TEM, and XRD. TEM analysis displayed that Ru metals dispersed well on Vulcan carbon with an average size of 1.98 nm. XRD analysis presented that the main phases in the ruthenium and Vulcan carbon correspond to an fcc structure. For electrochemical characterization, the enhanced effect of nanosensor was studied using CV and EIS techniques. In addition, scan rate, pH effect, nanomaterial amount, deposition time, and potential were also investigated for the selection of optimum conditions. As a result, the proposed new nanosensor showed enhancement for the oxidation peak current of IDA due to its improved electronic conductivity and electron transfer kinetics compared to bare GCE. The values of LOD and LOQ were calculated as 9.25 × 10^–9^ M and 2.8 × 10^–8^ M with a linear range among 5 × 10^–8^ M and 1 × 10^–6^ M. To demonstrate the applicability and validity of developed nanosensor, it was used for the determination of IDA in Idamen® IV (10 mg/10 mL vial) and human serum sample. The results of recovery studies showed that the Ru@VC/GCE nanosensor was free from excipient interferences in the dosage forms of injection, and it can be successfully applied to biological samples.
